# Systematic review of qualitative evaluations of reentry programs addressing problematic drug use and mental health disorders amongst people transitioning from prison to communities

**DOI:** 10.1186/s40352-018-0063-8

**Published:** 2018-03-02

**Authors:** Sacha Kendall, Sarah Redshaw, Stephen Ward, Sarah Wayland, Elizabeth Sullivan

**Affiliations:** 1 Australian Centre for Public and Population Health Research, Faculty of Health, University of Technology Sydney, Ultimo, NSW Australia; 2 Justice Health & Forensic Mental Health Network, Sydney, NSW Australia

**Keywords:** Prisoner reentry program, Qualitative evaluation, Health and welfare, Pre and post release planning, Support relationships, Structural factors

## Abstract

**Background:**

The paper presents a systematic review and metasynthesis of findings from qualitative evaluations of community reentry programs. The programs sought to engage recently released adult prison inmates with either problematic drug use or a mental health disorder.

**Methods:**

Seven biomedical and social science databases, Cinahl, Pubmed, Scopus, Proquest, Medline, Sociological abstracts and Web of Science and publisher database Taylor and Francis were searched in 2016 resulting in 2373 potential papers. Abstract reviews left 140 papers of which 8 were included after detailed review. Major themes and subthemes were identified through grounded theory inductive analysis of results from the eight papers. Of the final eight papers the majority (6) were from the United States. In total, the papers covered 405 interviews and included 121 (30%) females and 284 (70%) males.

**Results:**

Findings suggest that the interpersonal skills of case workers; access to social support and housing; and continuity of case worker relationships throughout the pre-release and post-release period are key social and structural factors in program success.

**Conclusion:**

Evaluation of community reentry programs requires qualitative data to contextualize statistical findings and identify social and structural factors that impact on reducing incarceration and improving participant health. These aspects of program efficacy have implications for reentry program development and staff training and broader social and health policy and services.

## Background

Effective community reentry programs are one component in strategies to reduce recidivism and assist in the successful transition of prison inmates to community. The rising rate of adult incarceration is a major public health and societal problem worldwide. Globally, there are an estimated 10.35 million people in custody (Walmsley 2015). Prison inmates are a vulnerable population characterised by complex health problems. People who have experienced imprisonment have higher rates of mental illness, infectious diseases, chronic diseases and mortality in comparison to the general population (Bradshaw et al. 2017; Fazel and Baillargeon 2011). Problematic drug use is pervasive, affecting approximately one third of male prison inmates and half of female prison inmates (Fazel et al. 2017). Inadequately treated mental health problems and substance use is associated with re-incarceration (Kinner and Wang 2014).

Reentry to the community is known to be highly stressful. This is attributable to the complexity of health problems and poor engagement with health and social services (Fazel and Baillargeon 2011; Fazel et al. 2006; Kinner et al. 2011; Kinner 2006). A recent systematic review of randomized controlled trials of community reentry interventions designed to improve prisoner health from imprisonment to 1 year post release concluded ‘the high burden of mortality, morbidity, and hospitalization post-release suggested that a greater focus on improving health in this population during and after release is warranted’ (Kouyoumdjian et al. 2015 p.e29) and that there are substantial gaps in evidence (also see Hayhurst et al. 2015).

We aim to identify and synthesise the factors relevant to successful community reentry identified by qualitative reentry program evaluations. A systematic review of the literature was conducted to synthesise current evidence in this area with a focus on reentry programs targeting mental health disorders and problematic drug use. People with mental health disorders and problematic drug use are over-represented in the prison system. This is in part due to a lack of community support services for these populations coupled with harsh legislation targeting particular behaviours (Brinkley-Rubinstein 2013).

The post-release period is a high-risk period characterised by poor continuity of care, inadequate social support and limited financial resources resulting in poorer health outcomes and a return to crime related activities (Binswanger et al. 2012). Prison inmates with a history of drug dependence are particularly vulnerable, with higher rates of morbidity, mortality and return to custody in the 6-month post-release period (McMillan et al. 2008) and a heightened risk for mortality in the first week post-release (Bukten et al. 2017). Prison inmates with a history of mental health disorder experience worse outcomes on release from prison including substance use, poor mental health, and criminal activity (Cutcher et al. 2014). Diagnosis of a major psychiatric disorder can be predictive of recidivism and associated with shorter time to re-incarceration (Fu et al. 2013). People with a dual diagnosis of mental health disorder and substance use have a risk of re-incarceration more than 40% higher than individuals with no diagnosis (Blank et al. 2014).

Recent research examining the causal relationship between problematic drug use and re-arrest shows a complex longitudinal association between these factors and identifies social factors such as access to support and services as significantly impacting these behaviours in the reentry population (Link and Hamilton 2017). This new evidence highlights the critical importance of access to effective reentry programs and social support for people exiting prison. Moreover, it indicates the relevance of qualitative program evaluation to understand the nuances of program efficacy and the particular quality and type of social components that program participants find beneficial.

Community reentry programs for people exiting prison are typically evaluated applying quantitative methods. Quantitative evaluation is necessary for examining program outcomes, however, when a program aims to improve the health of participants or prevent re-incarceration, quantitative methods are limited. Health is comprised not only of physical components that lend themselves to objective measurement but also subjective and relational dimensions that are embedded in participant experience. Similarly, the structural factors that impact on incarceration are more complex than statistical crime data can reveal (de Viggiani 2007). In contrast, qualitative evaluations of reentry programs reveal the experiential elements of program success and the social and structural aspects of reentry programs that impact positively on participant health and reducing incarceration. They allow for the exploration of the ‘why’ in program efficacy.

Detailed information gathered in qualitative evaluations can contribute to the demonstration of implementation intensity and program fidelity by connecting treatment subjects’ experiences with assessment of program elements (Miller et al. 2012; Miller 2014; Neale et al. 2005; Thomas and Harden 2008). Qualitative evaluations contextualize quantitative findings to effectively interpret a program’s holistic value and provide insight into source effects such as gender, ethnicity, education and other structural factors (CRD 2008). Synthesising qualitative research has similarly become important. Meta-synthesis seeks to explain and understand phenomena by pulling together findings from qualitative research (Stone and Seaman 2014) into a new integrative interpretation (Finfgeld 2003).

## Methods

### Sample and procedures

The aim of this review is to provide a synthesis of the factors relevant to successful community reentry identified by qualitative reentry program evaluations. The scope of the review was papers describing qualitative evaluations of reentry programs for people transitioning from prison to communities targeting substance use and mental health disorders.

A search of databases for published papers containing qualitative evaluations of reentry health programs was completed using the PRISMA statement in October 2016 with a cut-off date of 2006. The cut-off date was chosen as there were few published findings for such programs before this date. This was in part due to the fact that reentry programs are relatively new and also to the lack of evaluation of such programs (Lattimore and Visher 2013). The review was restricted to studies relating to adult prison inmates over 18 years of age.

**Fig. 1:**
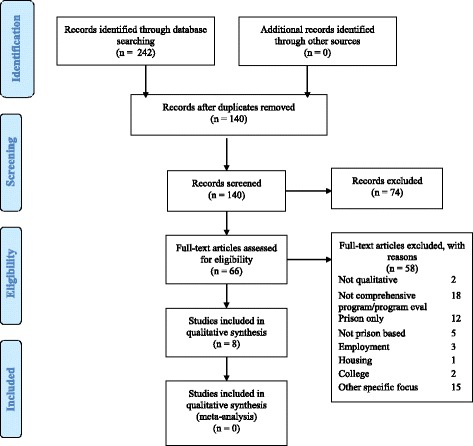
PRISMA flow diagram

The search included databases Cinahl, Pubmed, Scopus, Proquest, Medline, Sociological abstracts and Web of Science and publisher database Taylor and Francis. Search terms included ‘pre-release’, ‘transitional’, ‘reintegration’, ‘throughcare’ and ‘reentry program and prison’, ‘interview’ and ‘qualitative study’. Figure 1 details the search strategy in PRISMA format.

The search resulted in 2373 papers including duplicates. The most productive databases were Scopus, Proquest and Taylor and Francis, which yielded 2025 between them. Abstract reviews resulted in the selection of 140 papers. Of these, 66 appeared to relate prison inmates’ experiences of reentry programs. An iterative process then followed in which these papers were reviewed by the team of three researchers using a summary spreadsheet which included publication details, the focus of the paper and the abstract. Those relating primarily to substance use and mental health disorder were selected for review. This excluded papers focusing specifically on employment (*n* = 3), housing (*n* = 1) and college programs (*n* = 2) or specifically addressing a health condition, i.e. HIV (*n* = 1). The review then excluded those that were not qualitative (*n* = 2), not evaluating a program (*n* = 17), not concerned with prison inmates (*n* = 5) did not extend to community post-release (*n* = 12 focused on in-prison programs only), or were concerned with a specific group such as young or long term prison inmates (and did not meet the reentry health program criteria) (*n* = 16). See PRISMA diagram for reasons for exclusion and Table 1 for the final eight papers selected with characteristics of the studies involved.

**Table 1:** Final papers selected for detailed review

**Paper**	**Main issue**	**Name of program**	**Demographics**	**Method**
1. Angell et al. (2014). Engagement processes in model programs for community reentry from prison for people with serious mental illness. Int J Law Psychiatry, 37 (5), 490-500.	Mental illness	Critical Time Intervention (CTI) and Forensic Assertive Community Treatment (FACT)	Not given	37 Semi-structured interviews
2. Elison et al. (2016). Findings from mixed-methods feasibility and effectiveness evaluations of the “Breaking Free Online” treatment and recovery programme for substance misuse in prisons. Drugs-Education Prevention and Policy, 23 (2), 176-185.	Substance use	Breaking Free Online	Male, average age of 35.5 years (range 23–56 years) and all White-British	16 Semi-structured interviews
3. Gilbert and Elley (2015). Reducing Recidivism: An Evaluation of The Pathway Total Reintegration Programme. New Zealand Sociology, 30 (4), 15-37.	Services and support	Pathway Total Reintegration Programme	Male	12 Semi-structured interviews
4. Hunter et al. (2016), ‘A Strengths-Based Approach to Prisoner Reentry: The Fresh Start Prisoner Reentry Program’, International Journal of Offender Therapy & Comparative Criminology, 60 (11), 1298-314.	Services, case management	Fresh Start Prisoner Reentry Program Community Reentry Initiative	Males 64.5% Afr Am, 34% Latino	2 Focus groups × 12
5. Johnson et al. (2015). Development and Feasibility of a Cell Phone–Based Transitional Intervention for Women Prisoners With Comorbid Substance Use and Depression. The Prison Journal, 95 (3), 330-352.	Substance use, depression	Sober Network IPT	Female 19-54 years 4 Hispanic 3 Afr Am	22 Structured exit interviews
6. Miller et al. (2016), ‘Reentry programming for opioid and opiate involved female offenders: Findings from a mixed methods evaluation’, Journal of Criminal Justice, 46, 129.	Substance use	DCT program	Female 19-55 years	32 Semi-structured interviews
7. Pleggenkuhle et al. (2016). Solid Start: supportive housing, social support, and reentry transitions. Journal of Crime and Justice, 39 (3), 380-397.	Housing, social support	Solid Start program	Male, average age 40.8, 61% white	36 Semi-structured interviews
8. Zortman et al. (2016). Evaluating reentry programming in Pennsylvania’s Board of Probation & Parole: An assessment of offenders’ perceptions and recidivism outcomes. Journal of Offender Rehabilitation, 55 (6), 419-442.	Substance use	Pennsylvania’s Board of Probation & Parole (PBPP) 3 sites Berks County, Lackawanna County, York County	Male (86.2%, 79.3%, and 95.4%), young (41, 37, and 36%) slight majority from BC Hispanic (44.8%), majority LC White (86.2%), over half in YC Black (56.8%).	26 Semi-structured interviews
**Qualitative analysis methods**		**Findings**		
1. Constant comparative analysis frequently associated with grounded theory		Findings suggest efforts to bolster successful community entry relied heavily on efforts to help clients obtain resources through advocacy and side-by-side assistance. These efforts deemed the most useful for engaging justice-involved clients in the helping process.		
2. Interpretative phenomenological approach. Interview transcripts examined for quotes relevant to research aims, highlighted and notes made in margin on how quotes relate to research questions. Notes formed basis of themes identified and refined, with additional emerging themes added to the set.		Significant quantitative improvements to quality of life, severity of substance dependence and aspects of recovery progression illustrate initial effectiveness of BFO. BFO shows promise as one that can provide support that crosses the prison-community divide through providing continuity of care during the reintegration process.		
3. Interview data illuminated elements of the programme contributing to success or otherwise, and how. Semi-standardised questions with randomly-selected sample of clients. Nothing on analysis methods.		Quantitative data on 12-month recidivism rates of programme graduates show reoffending markedly reduced. Key finding from interviews - many different services were valued by participants depending on their individual needs, but consistent and highly individualised social work support was crucial.		
4. Two focus groups - one in prison, second in community at program site. Data analyzed for content and themes reflecting strengths based approach and highlighted challenges to approach and program implementation. Directed content analysis for themes related to the strengths-based approach and program identified by first author confirmed by the last author. Disagreements in themes discussed until agreement reached.		Focus group participants reported program followed-through to help them achieve their goals and was responsive to their needs, trust and respect for program staff and support experienced. Successful program strategies described included program culture, responsivity to needs, and the focus on strengths.		
5. Standardized, structured exit interviews with participants to ask about their perspectives on how to improve the intervention, when it was comfortable/uncomfortable and easier/harder to call study counselors, any barriers to being completely honest with study counselors over the phone, why women stopped calling if they did and suggestions for re-engaging them, and women’s thoughts about the schedule of the phone sessions, the counselors, and the phones themselves		Results suggest that providing contact with supportive, positive, familiar prison providers after release by giving women inexpensive cell phones is feasible, and that women perceive it as helpful.		
6. Results from both qualitative and quantitative data illustrate female participants’ outcomes as well as accounts of their offending, arrest, and incarceration. Quantitative data related to rearrest and probation violations were collected and analyzed to determine program effectiveness while qualitative interview data offer insights into offenders’ program experiences and their pathways to arrest and incarceration. (129-130)		1) Licit prescription drug use often predated transition into heroin and other opioid use; 2) Interpersonal romantic relationships played important role in pathways to substance abuse and criminal behavior; 3) Participants largely satisfied with the reentry program, staff, and treatment components, variable levels of perceived self-efficacy among the women; and 4) Some complaints of perceived gender-based inequities in programming and facility.		
7. Parolees asked about expectations for the future and their ability to be successful: ‘Where do you see yourself 1 year from now?‘4 Parolees in program asked additional questions about impact on reentry including perspectives on strengths and weaknesses of the program. Using NVivo analysis proceeded in three phases; grounded theory approach for initial analysis followed by focused coding to generate additional subthemes. Based on patterns related to housing, social support, and reintegration to the community.		Results suggest that provision of housing not only facilitated feelings of stability and independence, it also influenced cognitive shifts in commitment to change and hope for the future for those in the Solid Start group. In addition to housing, the importance of social supports via peer networks served as another social factor influencing subjective change.		
8. Content analysis techniques involved reviewing narrative accounts and initially coding into main themes. Specific subcategories were created from themes and enumerated. Discussion among evaluators and reaching consensus ensured reliability. Themes and categories related to substance abuse, cognitive distortions, and program staff were examined specifically.		Results suggest difference in opinion between offenders and service providers on identification of problems inside as well as outside correctional establishments. Highlights necessity to carefully assess support expectancies of incarcerated and released offenders, taking unique needs of each individual into account. Close respectful collaboration with clients and recognition as the main actors within their own treatment process condition for effective treatment.		

### Analysis

Following the most recent and well-cited protocol for qualitative reviews developed by Walsh and Downe (2006) all papers were reviewed by three team members for rationale, recruitment, methods of analysis and findings. The review included assessing the validity of results and for rigour across the rationale, methodology, findings and limitations. The final papers were examined in detail taking a grounded theory approach (Charmaz 2006) allowing for inductive analysis. Major themes and subthemes were identified through analysis of results from all papers. These themes were recorded in a table indicating the papers related to each theme. Detail from each paper relating to the themes identified were collected and summarised for each theme.

As in Thomas and Harden (2008), “going beyond” the content of the original studies was achieved by using the descriptive themes identified in inductive analysis of study findings. Barriers and facilitators inferred from the views participants expressed about reentry program experiences were captured in the descriptive themes and the implications of these views for reentry program development were then considered.

## Results

Of the final eight papers selected the majority (6) were from the United States, one was from the United Kingdom, and one was from New Zealand. In six studies it was stated that interviews were semi-structured, in one interviews were structured though there was opportunity for individual program participants to expand upon information gathered through the questionnaire by giving personal, narrative accounts, and one study used focus groups only. The number of interview or focus group participants ranged from 10 to 226. In total, the papers covered 405 interviews (including 24 in focus groups). Three papers focused on female participants only, four included males only and one included both. A total of 121 (30%) female and 284 (70%) male participants were included in the studies. Ages were provided as a range or as an average with a minimum 19 years and a maximum 56 years. One paper did not include information on age or ethnicity/race and three papers provided an age range but not ethnicity/race. In all studies participants were invited to be part of the research and voluntarily attended the program being evaluated. In three studies a carefully matched control group of those who chose not to or were unable to participate in the program due to their imminent release was used (Miller et al. 2016; Pleggenkuhle et al. 2016; Zortman et al. 2016). In one study, participants were initially randomly assigned by selecting every fourth name on a list of program participants over a five year period (Gilbert and Elley 2015).

The overarching conclusion from all papers reviewed was that participants in reentry programs benefit from a combination of practical resources and empathic support spanning from pre-release to varying lengths of time in the post-release period. Three major themes were identified across all papers: 1) structural context; 2) supportive relationships and 3) continuity of care including pre-release planning. The themes shown in Table 2 while distinct have some overlap and interconnection. The following discussion presents a metasynthesis of findings from the papers analysed following identification of common themes.

**Table 2:** Key themes identified with subthemes

Structural Context	Supportive Relationships	Continuity of Care
Housing	Prosocial network	Pre and post release follow through (continuity)
Employment	Professional support Psychosocial support Therapeutic support Nature of Program Advocacy Continuity of case manager relationship Practical assistance	
Stigma		Individual needs planning - substance use, health treatment, housing, employment etc. Recovery Capital Reintegration Prosocial identity Prosocial relationships Therapeutic relationship Community engagement Access to support and services Insight Coping skills Reconnection with family Ongoing informal support Gender difference
Individual needs/tailored support Pre-release planning Reconnecting with family		
Interpersonal relationships Provision of resources Linking to services Building rapport with case manager Peer support Personal agency Responsibility and independence Achieving goals Attitude change	Gender difference Case manager characteristics (advocate; non-judgementalism; trust; availability; reliability; respect; honesty; empathy; non-authoritative; supportive; solidarity; committed; care; motivating; knowledgeable) Responsibility and independence	

## Discussion

### Structural context

The importance of structural context was identified in all of the papers reviewed. The structural context, identified as the systems that govern an individual’s engagement with, or access to services includes aspects of the social system impacting on the individual’s health and wellbeing and capacity to avoid re-arrest. In the studies reviewed, structural context translated into specific forms of practical support provided via the program or relational and psychological factors associated with service provision.

Housing and employment were identified in all studies as the most critical forms of practical support or ‘recovery capital’ (Elison et al. 2016) in terms of desistance from problematic drug use and avoiding reincarceration (Gilbert and Elley 2015; Hunter et al. 2016). This was acknowledged in programs either through the direct provision of housing as in the Solid Start Program evaluated by Pleggenkuhle et al. (2016) or via the role of program case workers who were assigned to assist participants in accessing housing or employment by linking them in to other services (Angell et al. 2014; Gilbert and Elley 2015). In one study, housing was identified as the primary social factor impacting on reentry success and precursor for all other forms of social capital that might promote participant health and avoidance of re-arrest such as education, employment and pro-social relationships (Pleggenkuhle et al. 2016). In Zortman et al. (2016), progression through the reentry program was contingent on finding employment whilst obtainment of housing and increasing independence were seen as markers of reentry success. The Fresh Start program evaluated by Hunter et al. (2016) focused on building employment readiness, job seeking and retention in addition to coordination of housing and other community services. In Angell et al. (2014), case managers focused on assisting inmates with mental illness in building community connections with mental health services, housing/landlords and social networks in order to establish long term sources of support.

A significant issue for individuals exiting the prison system is how to overcome the structural deficits of stigma and discrimination associated with incarceration. Lack of credit history, finances, references from landlords and employment presented major barriers to participants securing housing post-release (Pleggenkuhle et al. 2016). A number of the studies highlighted the important role of caseworkers in assisting participants to overcome these structural barriers via advocacy and access to their knowledge and connections with services (i.e ‘social capital’) (Angell et al. 2014, p496; Gilbert and Elley 2015). Assisting program participants with accessing housing and other resources was also identified as an important way in which caseworkers bonded and established trust with participants (Angell et al. 2014; Hunter et al. 2016; Pleggenkuhle et al. 2016), which in turn promoted personal agency and attitude change in participants (Pleggenkuhle et al. 2016). These factors are identified as relevant to both positive reentry experience and success in the short term but also capacity to reintegrate into the community in the longer term through access to resources and sustainable social support (Elison et al. 2016; Gilbert and Elley 2015).

The combination of both resources and empathic support provided by caseworkers produced positive relational and psychological outcomes for participants in the short and long term including reconnection with family (Hunter et al., 2016), improved interpersonal relationships (Zortman et al. 2016) improved self-efficacy (Pleggenkuhle et al. 2016) and formation of pro-social identity (Gilbert and Elley 2015). Pleggenkuhle et al. (2016) note that stable housing was associated with increased personal agency, optimism, goal-setting, success and responsibility. Participants experienced changes in attitude and thinking and were more likely than the comparison sample to describe future plans such as specific career paths or more definite educational or vocational plans and to demonstrate attitudinal changes (Pleggenkuhle et al. 2016, p.390). The Pathway program evaluated by Gilbert and Elley (2015) included community volunteering to reintegrate participants in their local communities and link them to pro-social activities. Participants reported that this experience promoted positive psychological changes that were protective against recidivism including development of a pro-social identity through the overcoming of negative stereotypes, contribution to the community, responsibility and new pro-social relationships.

In all studies, participants reported the benefit of case managers working to understand and meet their individual needs. This was attributed to the action taken by case managers but also the disposition and commitment of case managers to provide clients with help and support. Case managers’ efforts to tailor support to their individual clients was highly valued by participants and central to program efficacy in terms of addressing the causes of ongoing offending (Gilbert and Elley 2015, p 20). Participants reported that program responsiveness to individualised needs and goals resulted in connection to relevant resources and improved their chances of reentry success (Hunter et al., 2016). The characteristics of supportive relationships reported by program participants are outlined in detail in the next section.

### Supportive relationships

The interpersonal skills of program case managers were identified as central to program efficacy. Characteristics such as empathy, honesty, non-judgmentalism, perseverance, reliability, care and commitment were repeatedly cited by participants as factors that contributed to their success in the program and reentry experience. Participants spoke of case managers as highly supportive (Gilbert and Elley 2015, p25-26) and going ‘above and beyond’ for them (Angell et al. 2014, p494). In Johnson et al. (2015) counsellors were recognised as a dependable support person during the reentry period when participants were feeling anxious, lonely or stressed (p344).

These characteristics engendered particular qualities in the client relationship. Participants described relationships with case managers built on trust, openness, respectful communication, solidarity and support. Trust was especially important for participants with serious mental illness because of participants’ past interactions with authorities, which were often coercive in nature (Angell et al. 2014, p493). In addition, case managers were valued for being knowledgeable, non-authoritative, hopeful, persistent and available in a crisis. Participants reported how beneficial it was for them to have someone who didn’t give up on them (Gilbert and Elley 2015), someone who was there when they needed them (Johnson et al. 2015) and someone who had the knowledge and persistence to advocate on their behalf and assist them in overcoming structural barriers to reintegration (Angell et al. 2014; Pleggenkuhle et al. 2016).

Where bonding was established with the case manager, participants were motivated to succeed in the program and described the relationship as fostering their responsibility and independence (Gilbert and Elley 2015; Pleggenkuhle et al. 2016; Zortman et al. 2016). Participants had a vested interest in the program because of the alliance and ongoing relationship they had with program staff (Johnson et al. 2015). Effective engagement with clients through positive communication and practical assistance promoted investment in the shared tasks of treatment and enabled staff to provide client-centred care (Angell et al. 2014). In some instances the support of caseworkers was directly credited for keeping the participant out of prison (Gilbert and Elley 2015, p23).

The theoretical and methodological underpinnings of programs are also relevant, as these influenced the nature of the client relationship and program focus. For example, in Angell et al. 2014, acknowledgement that prison inmates with serious mental illness are most likely to prioritise reconnection with informal networks on release from prison meant that case managers coordinated their efforts with clients’ primary network members (Angell et al. 2014, p 495). Similarly, clients in the Fresh Start Prisoner Reentry Program noted that case manager contact with family members helped to solidify reentry plans (Hunter et al. 2016, p1306). The online model of transitional care evaluated by Elison et al. (2016) focused on developing the recovery capital of inmates by building individual coping skills. Participants in this program reported benefit from developing these skills but also significant anxiety about desistance from problematic drug use and crime in the post-release period without therapeutic and practical support.

The programs evaluated by Gilbert and Elley (2015) and Hunter et al. (2016) were both underpinned by the ‘Good Lives Model’, which focuses on meeting individual needs and promoting long term reintegration via a strengths-based approach. This model is built on the premise that ‘risk can be managed by promoting knowledge, strengths, skills and access to internal and external resources’ (Hunter et al. 2016, p1301). In both these studies clients reported the benefit of ongoing support from supportive case managers who worked with them to meet their individual needs and goals. This included access to resources and connection to social networks and community activities that were protective against re-offending.

The importance of pro-social relationships was noted in several studies. Gilbert and Elley (2015) highlight the benefit of peer and mentor relationships in terms of building pro-social identity and support networks. Zortman et al. (2016) identify prosocial support networks as reinforcing pro-social behaviour and an essential element in the rehabilitation and reintegration of offenders with a history of problematic drug use. Pleggenkuhle et al. (2016) note the motivational and therapeutic benefits of sharing experiences with peers facing similar challenges (Pleggenkuhle et al. 2016, p389). In recognition that women needed assistance to initiate contact with positive sober people in the community, the Sober Network Interpersonal Psychotherapy (IPT) program included interventions to build communication skills and connect participants with a sober support network (Johnson et al. 2015, p336).

The papers evaluating female programs (Johnson et al. 2015 and Miller et al. 2016) identified particular social factors impacting on program efficacy. These factors were relational. Miller et al. (2016) found that women’s pathways to problematic drug use and crime are strongly associated with romantic partner relationships. Women also reported pathways to addiction and offending resulting from childhood trauma. These factors are relevant to program efficacy because even when women were engaged in the program and held positive views about rehabilitation, women experienced significant self doubt about their capacity to stay sober during the post-release period (p132). Women also reported significant dissatisfaction with perceived gender inequities related to treatment and health care in prison (p133). Significantly, these findings were not revealed by the quantitative component of the program evaluation, highlighting the importance of qualitative program review. Miller et al. (2016) conclude that reentry programs need to be developed including clearly definable female-specific components such as trauma-informed care and based on further understanding about choices related to social networks and relationships (p134).

Johnson et al. (2015) similarly argue that an interpersonal approach to Substance Use Disorders (SUD) and Major Depressive Disorder (MDD) is imperative to meeting the needs of incarcerated women because interpersonal difficulties not only affect MDD but are also predictors of SUD relapse and recidivism in women (p331). This program was a relationship-based intervention including ongoing therapeutic support from a prison counsellor and assistance with building a supportive peer network. Participants in the study reported that access to a structured program post-release was beneficial because of their lack of prosocial relationships with people who are sober (p344). The therapeutic relationship was especially important to staying sober because women highly valued the continuity of care from a familiar and trusted professional. Counsellors could effectively engage women in prison and post-release, even in instances of relapse (p345).

Continuity of care from the *same worker* made a significant difference to the women in the Johnson et al. (2015) study, however, continuity of care was a primary theme identified across all papers. The following section will outline this in further detail.

### Continuity of care

Continuity of care is essential to building ‘recovery capital’ that extends beyond short term reentry to long term reintegration (Elison et al. 2016). As outlined above, ‘recovery capital’ took various forms in the studies reviewed including resources such as housing and employment; pro-social relationships; pro-social identity; coping skills; and community engagement. Continuity of care provided by professional staff allowed for attention to individual needs and the formation and maintenance of a therapeutic relationship (Hunter et al. 2016; Zortman et al. 2016). In Hunter et al. 2016, program services were even added over time in response to participants’ stated risks, needs, strengths and goals (p1308). Participants identified this responsiveness as a key strength of the program. In Zortman et al. (2016) participants cited the continuity of care from case managers as an important component in their progression through the program, sustained success and relapse prevention. Through continuous support, participants were able to develop insight into their problems and build skills and resources to prevent substance use relapse (Zortman et al. 2016). Johnson et al. (2015) also report a significant decrease in depressive symptoms and substance use amongst program participants from baseline to 3 months post-release, attributed to the continuous support provided by counsellors.

Other participants reported that through ongoing case manager support and resources they were able to change their lives and experienced increased independence and responsibility over time as a result of these supports (Gilbert and Elley 2015). In some studies this was enhanced by supportive peer and family relationships (Angell et al. 2014; Gilbert and Elley 2015; Pleggenkuhle et al. 2016; Zortman et al. 2016). Indeed, due to the limited resources in some studies, there was a focus on reconnecting participants with family or new social supports in order to build sustainable recovery capital (Angell et al. 2014).

Pre-release support was identified as critical to success in the post-release period in terms of initial identification of needs and goals and building rapport with case managers (Angell et al. 2014; Hunter et al. 2016; Miller 2014). Pre-release planning was administered in differing ways across the programs. Elison et al. (2016) found potential for an online program to support the process of recovery from substance use in prison and provide continuity of care in the reentry process. Elison et al. (2016) identify graded transition from prison to community with enhanced opportunities for intervention and rehabilitation as possible contributors to ‘increased effectiveness and sustained therapeutic benefits’ of the program (p.177). Another program, Sober Network Interpersonal Psychotherapy, ‘provides contact with the same prison-based counsellor from within prison through the first 3 months after release to stabilize women until they can get established with community treatment providers’ (Johnson et al. 2015, p332). Johnson et al. (2015) give accounts of pre-release program processes such as establishing positive social connections by reaching out to sober people while still incarcerated.

The Solid Start housing provision and social support program included pre-release planning which enabled physical separation from prior residence and opportunity for change, case management and coordination of services according to individual need (Pleggenkuhle et al. 2016, p.383). The Fresh Start program evaluated by Hunter et al. (2016) included case managers working with their clients to complete a ‘ReEntry Plan’ reflecting the clients’ strengths, goals, and identified needs so that each participant had a treatment plan before exiting the correctional facility. Case managers in the Fresh Start program ‘served as a natural bridge for individuals transitioning from prison to … community’ and sought to (a) enhance motivation and engagement; (b) establish clear collaboration between the criminal justice system, treatment providers, and community supports; (c) establish continuity of care; and (d) provide pre- and post-release supports (p.1303).

Program staff in the study of Miller et al. (2016) also assisted program participants through individualized ‘Reentry Accountability Plans’ including coordination of mental health, medical, and drug treatment and linking to community resources prior to release’ (p.131). In the Pathway Program, individual needs are assessed in a 2-month ‘phasing in’ pre-release process reported by participants as important to preparing for release and decreasing the stressors associated with reentry (Gilbert and Elley 2015, p.24). Participants reported finding value in different service elements, reflecting the individualised nature of the client plans (Gilbert and Elley 2015).

Program participants recognise the need for ongoing support and in some instances, the opportunity for continuity of care was the primary motivator for program participation (Hunter et al. 2016; Johnson et al. 2015). Participant motivation is an important factor in program efficacy, as Pleggenkuhle et al. (2016) demonstrate that the facilitation of positive and practical attitudes can be an important mechanism of desistance (p393). Overall, participants across all studies reported that continuity of care offered the tools to become independent. This was not only about accessing housing and other structural resources but the psychological shifts that come through ongoing support and building of recovery capital.

## Limitations of the studies

There are common limitations across all of the studies reviewed. These are typical of qualitative research and include: 1) Small sample sizes; 2) Unable to establish causality because the focus was on eliciting participant experiences; and 3) Non-generalizability of findings. Non-generalizability of findings is relevant in that most of the studies are from the United States and results may not translate elsewhere due to differences in prison systems but also applies at the program level, i.e. the experiences of one set of participants cannot be generalised to other potential program users.

Selection bias is also a limitation across many of the studies. For example, Pleggenkuhle et al. (2016) note that participants in their study may have been more motivated than other inmates. Hunter et al. (2016) recognise that their study lacked a comparison group and Miller et al. (2016) identify that their control group was not randomized. There is a need for more longitudinal data (Elison et al. 2016; Gilbert and Elley 2015; Hunter et al., 2016) and more data on female reentry experience (Angell et al. 2014). Gender issues related to program content were raised in only two of the papers reviewed (Johnson et al. 2015 and Miller et al. 2016). Moreover, the findings of Johnson et al.’s (2015) study are limited, as they were unable to establish causality because participants were also engaged with mental health and substance use disorder treatment services. Zortman et al. (2016) identify that there are limitations to the validity of their data because participant responses were self-reported and there was a lack of standardization across their research sites.

Description of method of analysis varied between papers and in one case was absent (Gilbert and Elley 2015). It was evident nevertheless that in all cases a variation of thematic analysis was used, which is arguably the most common method of initial analysis in qualitative research (Braun and Clarke 2006). Some papers then applied a theoretical approach to the discussion of the analysis such as an interpretative phenomenology (Elison et al. 2016) or a strengths based approach (Hunter et al. 2016). Greater uniformity in qualitative evaluation methods is desirable in bringing together results from similar studies.

## Limitations of the review

There is limited qualitative program evaluation research available and standardization of qualitative evaluation and inclusion is a work in progress. There were some differences in programs that have not been examined in detail in our analysis though we did find similar elements in the program evaluations included. Data analysis methods applied in the qualitative evaluations reviewed were forms of thematic or grounded theory analysis. Our systematic review analysis has treated all methods similarly and focused on reported results. More consistency is needed in qualitative methods used in evaluation studies to enable comparisons and review of the literature.

## Conclusion

This is the first systematic review of qualitative evaluations of reentry programs. Findings suggest that access to social support, housing and employment; the interpersonal skills of case workers; personalized approaches to case management; and continuity of care throughout the pre-release and post-release period are the key social and structural factors in program success. These factors impact on other measures of program efficacy such as reduced substance use and protecting against re-incarceration. The role of caseworkers as an advocate and advisor for program participants plays an important role in program success, as respectful communication combined with practical support was identified as beneficial in all papers. For women, the relational aspects of caseworker support such as trust and rapport are critical to program participation and relapse prevention. Continuity of individualised care goes some way to addressing the risk factors associated with reentry by assisting clients in establishing ‘recovery capital’. This includes accessing and maintaining housing and employment and providing an ongoing therapeutic relationship and connection to pro-social relationships. Where sustainable recovery is achieved, this can be transformative, resulting in reintegration into the community, long term desistance from substance use and crime and improved psychological health (Gilbert and Elley 2015).

## Implications for public policy

The review indicates that comprehensive reentry programs that address the full range of social and structural issues via individualised support from case managers can be effective. Participants report benefit from reentry programs where a combination of practical resources and empathic support is provided spanning from the pre-release to the post-release period. The need for integrated, rather than crisis-driven support, and gender specific health and social support services to support reentry is also indicated.
